# Cases from Dr. Hamilton’s Clinic at the Buffalo Hospital of the Sisters of Charity

**Published:** 1859-02

**Authors:** J. Boardman


ART. IV.—*Cases from Dr. Hamilton's Clinic at the Buffalo Hospital of the Sisters of Charity.* Reported by J. BOARDMAN, M. D.*

*Encysted Tarsal Tumors.* Sarah C., aged sixteen years, was brought before the class, having a small encysted tumor upon the upper lid of the left eye. Upon examination, there was seen a small, movable, well defined tumor, nearly round, and about three-eighths of an inch in diameter. It had never been painful. To both the eye and finger, it appeared much as if a pea had been introduced under the skin upon the cartilage of the lid. This was the second tumor of the kind she had had within a year. Dr. Hamilton removed the first, a few months since, of which a slight scar only remained to mark the spot.

Dr. Hamilton operated, by cutting with a bistoury, in a line parallel to the edge of the lid, through the skin and the external wall of the tumors, the contents, which were fluid, escaped, and then he removed most of the sac. It was dressed with a piece of dry lint.

These tumors are frequently met with upon either lid, lying anterior to the tarsal cartilage. They rarely, if ever, exceed one-half of an inch in diameter, and, if left to themselves, generally open and remain fistulous, the

---

*CORRECTIONS.—In the last number, page 464, line 18th, please read *the* interrupted sutures, instead of *his* interrupted sutures; also page 465, line 23d, read, the late *Dr. Horner,* in the place of *Dr. Homer;* also page 466, line 4th, read Ambrose *Pare,* not Ambrose *Raze.*

walls of the sac secreting a fluid, which runs out upon the cheek to the annoyance of the patient.

The cure is accomplished by destroying the sac, either by removing the entire tumor, or a portion of its walls, trusting to inflammation to destroy the secreting power of the part remaining. Some advise that the lid should be everted, and the tumor punctured through the cartilage, but generally the sac will be filled again in a short time. It is not always easy to remove the tumor, or even to open it with a knife, for, situated as it is in the loose tissue of the lid, it slides from under the edge of the knife, it being difficult to hold it firmly. This has led to the invention of an instrument, consisting of two plates, one of which is perforated; the lid is grasped between these plates, and the tumor projecting through the opening is thus held firmly, and can be easily removed.

*Nasal Polypi.* Wm. P., aged sixteen years, was brought before the class. For the last eight or nine months, he had difficulty in breathing through the right side of his nose, and for the last few weeks, the right nostril has been almost entirely closed. A large, fleshy tumor could be seen upon examination, filling the right nostril, and the probe discovered that it was attached by a broad neck, or base, to the superior maxilla, in front and below the opening of the antrum, that it filled the cavity of the antrum, and that it was rather firm in its consistence. His physician, six weeks before, had attempted to remove it by means of the forceps, but abandoned the operation on account of the excessive hemorrhage which took place. The trouble has increased since that time.

Dr. Hamilton attempted to remove it, by seizing the base with a strong pair of polypus forceps, making traction, at the same time rotating the instrument. In this way, he tore the tumor from its base, drawing it from the cavity of the antrum with comparative ease, for there were no attachments within the walls of the antrum, but the tumor seemingly filled the cavity, as the result of mechanical pressure. He then introduced the forefinger of the right hand through the external opening of the nostril, and with the finger-nail, scraped as clean as he could, the surface of the bone, where the polypi had been attached. The hemorrhage, which had been very free, through the whole operation, upon the withdrawal of his finger increased, so much so, as to threaten the immediate life of the patient. He re-introduced his finger, which, in part, controlled the bleeding, while an assistant prepared a plug, and obtained a flexible catheter; for this *great hæmorrhage* was unexpected, and he did not have the instrument (Belloc's sound) for plugging the nostrils, with him. The plug was composed of a roll of patent lint, about half of an inch in diameter and two inches in length, covered with ceratum simplex, and tied about its center with three long, strong, silk threads. The catheter was introduced through the nostril, and the end brought out of the mouth, by means of a pair of forceps. Two of the threads were fastened to this end, and by means of the catheter, they were brought out of the nose, and the plug, being guided by the finger carefully over the soft palate, was drawn firmly into the posterior opening of the nares, completely closing it. Dr. Hamilton did not wait for a plug to be made for the anterior opening, but stopped it up with pieces of lint, forced up with his probe. Generally, he uses a plug like that at the posterior, for the anterior opening, around which the two strings are firmly tied, and each plug, thus holding the other in place, prevent all further hemorrhage. The third thread, the object of which is to withdraw the posterior plug, was brought out of the mouth, and the end fastened upon the cheek, by a piece of adhesive plaster. The patient was then placed in bed. The fourth day Dr. Hamilton removed the pieces of lint from the anterior opening, and attempted, by means of the thread carried over a finger, introduced behind and below the soft palate, to withdraw the posterior plug, but it was so firmly wedged that the thread broke. As there was a slight hemorrhage, it was left for three days longer; then the plug was grasped with a pair of forceps, and drawn gradually forward through the anterior opening. In a week the patient left the hospital, feeling free of his trouble, the truth of which, time alone can prove.

Polypi are the offspring of mucous membranes, and the name has been applied to all bodies, or tumors, hanging from them. They are spoken of by different writers, as being most frequently found in the nose.

Nasal polypi are not confined to any particular age, but may be found at all periods of life, though I do not recollect ever to have seen or heard of an instance in a child under ten years of age.

It is difficult to determine the cause of their production; generally, their appearance has been preceded by at least a feeble condition of the general health, though sometimes they occur in patients who seem to be perfectly well. Mr. Abernethy, in his lectures at St. Bartholemew's Hospital, states that "every disease connected with the pitutiary membrane, is connected with the state of the constitution;" and he states a case of a man who was in the habit, for a number of years, of having polypi removed from his nose. One day, examining him more closely, he discovered that his stomach was "all wrong." To use his own words: "I gave him a lecture about his bowels and from that time he never had a polypus extracted from his nose for many years. Several years after, he fell down, as I believe, on the neck of his thigh-bone, and he was laid up for a long while; after that, he once called upon me to have the polypi extracted again." It is but fair to state, that by Mr. Abernethy, the bowels were accused of being the cause of almost every disease.

Dr. Hamilton classes nasal polypi as follows:

1st. Gelatinous, or oyster polypi.
2d. Hydatid, or muco-cystic.
3d. Fibrous, or fleshy.
4th. Carcinomatous.
5th. Fungoid.

Of these, the gelatinous, or oyster polypi, is, by far, the most frequent. "It is composed of the elements of the mucous membrane, expanded and spread out, and consists of a loose fibrous stroma, covered with epithelium." They grow from a narrow pedicle, are but very slightly vascular, and have a pale, grayish yellow color; growing often to a considerable size, so that they may be seen in the throat, hanging below the soft palate. Not unfrequently several exist at the same time.

The hydatid polypi are formed by a collection of hydatids, and appear like a collection of bags of water. Sir Astley Cooper taught that this variety gen erally was to be found in young persons. It is, however, a rare form, and in twenty-five recorded cases, in Dr. Hamilton's notes, I find but one case of the hydatid variety.

The fibrous polypus consists of a fibrous or fleshy tumor, covered with mucous membrane, growing from some of the bones of the nasal cavity, firm in its consistence, generally attached by a broad base, vascular, and liable to bleed freely from slight causes. This, like the two first varieties, originates without pain, and may never be the seat of much pain, unless it degenerates into the carcinomatous, or fungoid variety, which sometimes occurs.

The cacinomatous and fungoid variety, have the general character and appearance which those structures present in other parts of the body, and, like those tumors, equally resists curative treatment.

The two first forms are comparatively harmless, except that occasioned by mechanical pressure, never, I believe, degenerating into the malignant polypus. They are frequently entirely destroyed by an operation; yet, sometimes, returning again and again, year after year. The third variety, however, is a more serious malady, endangering life, not only by profuse and frequent hemorrhage, but by pressure, exciting caries of the surrounding bones, and also by its tendency to degenerate into the carcinomatous, or fungoid variety. In its early stages, it is often removed with success, but it is exceeding prone to return, unless its base is entirely destroyed.

John Bell regarded all forms of polypi as of one and the same variety. He writes: "Polypus is never mild, nor even malignant; time, and the natural growth of the tumor, and the pressure it occasions within the soft and bony cells of the nostrils and fauces, must bring every polypus to one invariable form, in its last and fatal stage." He regards the early stages as curable, but as soon as the polypus becomes painful, he declares it to be incurable.

The symptoms which attend the formation of polypi are much the same as those attending a common cold. There is present sneezing, and some irritation of the nostril, snuffing, and a mucous discharge from the nose, a sense of fullness of the head, especially in damp weather, sometimes the tumor can be felt to move backwards and forwards with the expiration and inspiration. The voice becomes changed, the eyes are filled with tears, and the hearing is impaired from pressure upon the eustachian tubes; a sense of fullness and headache follow, the discharge becomes exceedingly offensive, and, at times, the patient is willing to submit to anything that promises the least relief.

To effect a cure, various means have been used, astringent and caustic applications, etc., but only to be given up as useless. The mechanical removal of the polypi is now held to be the only plan that offers fair prospects of accomplishing a permanent cure. To effect this, the ligature and forceps have both been used. Various instruments have been invented for passing the ligature around the neck of polypi, and both wire and silk, have each had their advocates, but at present the ligature is comparatively rarely used, save for a polypus which is attached far back in the nostril, the body of which may be seen hanging, below the palate, in the throat. But the removal, by the forceps, is the plan in most general use, and has proved the most effectual. It is accomplished, as described in the case at the hospital, by grasping the neck of the polypus with a pair of forceps, the blades of which are serrated, and making gentle traction, at the same time rotating the instrument; in this way, the whole base is thought most likely to be removed. The forceps, at times, will bring away, with the base, a thin plate of bone to which it was attached; but this is a matter of little moment, and by many is considered as good evidence of the complete removal of the tumor.

This last plan is the one of which Mr. Abernethy said: "It is as blackguard and unscientific an operation as any I know; but yet it is the only one I know of that will answer the purpose."

There was one part of the operation which has been described, to which Dr. Hamilton particularly called the attention of his class; the introduction of the finger into the external opening of the nose, and removing with the nail all that remain of the attachments of the polypus. This, at first sight, seemed impossible, but this was the second time that he had used his finger in that way. The first time, the patient was a lady, on whom he was operating for polypus. In the operation, it was difficult to remove the attachment as completely as he wished, and trying with his fingers, to see if he could not reach the point, he found, to his surprise, that he could introduce with but little trouble, his whole finger into the nose. He would not recommend that it should be done in every instance, but he wished the class to note that it could be done, if, at any time, there should arise a necessity for it.

In looking over twenty-five cases in Dr. Hamilton's notes, I find they are comprised of nineteen cases of the gelatinous variety, one case of the hydatid, and five cases of the fibrous, or fleshy polypi. He states he has never seen an instance of the carcinomatous, fungoid variety. In three instances, all of the fleshy variety, the polypi were not removed, and of the others, only four were removed with the ligature, fifteen were removed by the forceps, and, in three cases the method was not mentioned. The bleeding was slight, except in three instances, including the case mentioned in this paper, this case, also, being the only one in which it did not cease in a few moments of itself. In two of the cases not operated upon by removal of the polypi, the external carotid was tied on account of the great and frequent hemorrhage from the tumor. In both instances the tumor diminished in size, and, for a time, the bleeding entirely ceased; and though, after a time, it was the cause of death, yet, in one instance, much time, more than a year, was seemingly added to the life of the patient.

